# c-MET Protects Breast Cancer Cells from Apoptosis Induced by Sodium Butyrate

**DOI:** 10.1371/journal.pone.0030143

**Published:** 2012-01-12

**Authors:** Bo Sun, Rui Liu, Zhong-Dang Xiao, Xuan Zhu

**Affiliations:** 1 State Key Laboratory of Bioelectronics, School of Biological Science and Medical Engineering, Southeast University, Nanjing, China; 2 Laboratory of Biophysics, School of Biological Sciences, Seoul National University, Seoul, South Korea; 3 School of Pharmaceutical Science, Xiamen University, Xiamen, China; National Cancer Center, Japan

## Abstract

Sodium Butyrate (NaBu) is regarded as a potential reagent for cancer therapy. In this study, a specific breast cancer cell population that is resistant NaBu treatment was identified. These cells possess cancer stem cell characters, such as the capability of sphere formation *in vitro* and high tumor incident rate (85%) in mouse model. Forty percent of the NaBu resistant cells express the cancer stem cells marker, the CD133, whereas only 10% intact cells present the CD133 antigen. Furthermore, the endogenous expressing c-MET contributes to the survival of cancer stem cell population from the treatment of NaBu. The CD133+ group also presents a higher level of c-MET. A combination treatment of MET siRNA and NaBu efficiently prohibited the breast cancer progression, and the incident rate of the tumor decrease to 18%. This study may help to develop a new and alternative strategy for breast cancer therapy.

## Introduction

Sodium butyrate (NaBu) is the sodium salt of butyric acid produced by large intestinal micro flora. As a potent histone deacetylase (HDAC) inhibitor *in vivo*, NaBu has been reported to regulate a large number of genes in cultured mammalian cells [1]. It has been reported that the NaBu induce growth arrest, apoptosis or differentiation on different cancer cell lines, including breast cancer cell lines [2]. Since it exhibits cancer chemotherapeutic potential, NaBu has been considered as a potential regent for cancer therapy [3]. Recently, It was reported that NaBu induces breast cancer cell differentiation by regulating β-casein and N-myc downstream-regulated gene 1 (NDRG1) in breast cancer cells [4]. Caspase-10 also plays an important role in the induction of apoptosis by NaBu on breast cancer cells [5]. However, cancer is composed of heterogenerious population of cells and the function of NaBu on different population needs to be elucidated.

*MET* oncogene, encodes for the tyrosine kinase receptor for hepatocyte growth factor, has been shown to be over expressed in various type of tumor cells [6], [7]. It contributes to the invasive growth of cancer cells through hepatocyte growth factor paracrine stimulation. Within the intracellular portion of MET, Tyr1234 and Tyr1235, mediates *MET* biological activity and the key tyrosine residues in the carboxy-terminal tail, Tyr1349 and Tyr1356 capable of recruiting downstream adapter proteins with Src homology-2 (SH2) domains [8]. Several oncogenic pathways are recruited by the engagement of MET, including Ras-Erk/mitogen-activated protein kinase (MAPK) pathway, Rac1/Cdc42-PAK pathway and Gab1-phosphoinositide 3-kinase (PI3K)-Akt pathway [8], [9], [10]. Moreover, c-MET may work with WNT or NOTCH pathways for the self renewal of cancer cells or stem cells [11].

In breast tumor patient, the relationship between c-MET up-regulation and tumor progress has been demonstrated [12]. Studies revealed MET overexpreesion correlated with aggressive phenotype of different cancer, including breast cancer. Indeed, c-MET has been regarded as a novel target for therapeutic approaches because of the significant correlation between c-MET overexpression and a high risk of disease progression [13], [14]. However, the interaction between c-MET and NaBu, the HDAC inhibitor is not clear. In the present study, we found that c-MET protects breast cancer cells from the apoptosis induced by NaBu. Moreover, we also demonstrated that a NaBu- resistant population expressed a high level of c-MET with cancer stem cells property. The result also indicated that, although regarded as a tumor suppressor, NaBu might be not sufficient to remove cancer stem cells and to prohibit the recurrence of breast cancer.

## Results

### Anti-tumor efficiency of NaBu was different in MDA-MB-231 cells and MCF-7 cells

**Figure 1:**
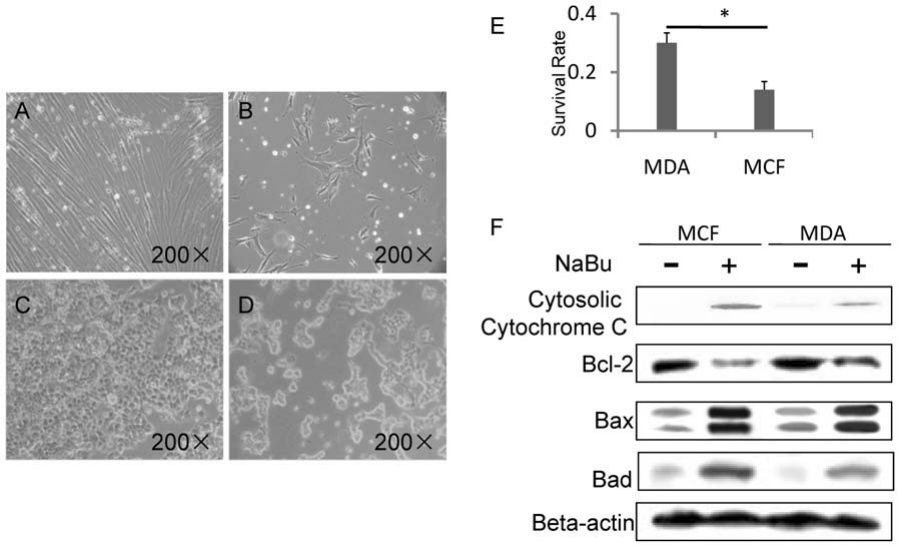
NaBu induced tumor suppression effect was dependent on cancer cell types. (A) to (D) showed the effect of NaBu on breast cancer cell line MDA-MB-231(A and B) and MCF-7 (C and D). Majority of MDA-MB-231(B) and MCF-7(D) cancer cells were removed by the treatment of NaBu. Compared with 16% of viable MCF cells, the survival rate of MDA-MB-231 was significantly increased to 30%(E). Immunoblotting result of BAD, BAX, Bcl-2 and Cytochrome C expression indicated that MDA-MB-231 cells were resistant to the treatment of NaBu by neutralizing its apoptosis effect compared with MCF-7 cells (F). *P<0.05.

In order to test the tumor suppression effect of NaBu in different types of breast cancer, two kinds of breast cancer cell lines, the estrogen receptor negative MDA-MB-231 and the estrogen receptor positive MCF-7, were rendered the treatment of different concentration of NaBu (1–5 Mm, data not shown) for 2 days. It was observed that the MDA-MB-231cell line showed a better survival rate than the MCF cell line (Fig. 1A–D). Since the most significant different survival rate between the two cell lines presents under 4 mM Nabu treatment, we applied this concentration for studies thereafter. Nearly 30% of MDA-MB-231 cells remained viable compared with only 16% of MCF-7 cells survived under the same condition when treated with 4 mM Nabu, as assessed by MTT assay (Fig. 1E). The 4 mM Nabu treatment showed the most significant survival rate between the two cell lines, therefore, we applied this concentration for further studies

Since previous studies demonstrated that NaBu induced cell apoptosis in MDA-MB-231 and MCF-7 [15], [16]. Herein, we investigated whether the different survival rate between these two cell lines is caused by their different response to the treatment of NaBu. Indeed, western blotting results showed higher expression level of Bad, Bax and Cytosolic Cytochrome C, but weaker expression level of Bcl-2 in MCF-7 cells than in MDA-MB-231 cells after NaBu treatment (Fig. 1F and Figure S1). These results suggested that the MDA-MB-231 cells were more resistant to apoptosis effect after NaBu treatment as compared to MCF-7 cells. By knocking down the expression of Bad or Bax, the apoptosis effect of NaBu was attenuated in MDA-MB-231 cells (Figure S2). These results suggested that the NaBu caused the MDA-MB-231 cells loss by apoptosis induction effect.

### c-MET contributed to the survival of breast cancer cells after the treatment of NaBu

**Figure 2:**
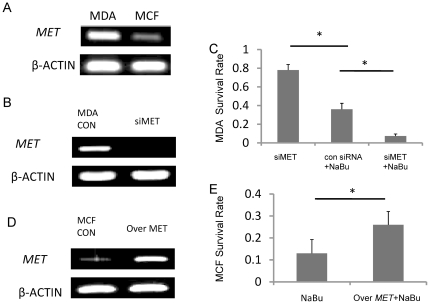
c-MET helped the survival of breast cancer cells after the treatment of NaBu. RT-PCR result illustrated that the expression level of *MET* was higher in MDA-MB-231 cell than in MCF-7 cells (A). The expression level of *MET* in MDA-MB-231 cells were decreased by the treatment of MET siRNA (B). Silencing *MET* in MDA-MB-231 cells resulted in a poor survival rate (7%) when the cells were treated by NaBu (C). For MCF-7 cells, while the *MET* expression was increased obviously by induction of *MET* activator (D), the cell survival rate were increased significantly with the presence of NaBu in culturing medium (26%)(E). *P<0.05.

Studies have illustrated that *MET*, an oncogene that promotes the progression and invasion of cancer cells, contributes to the cell proliferation by minimizing the apoptosis [7]. We demonstrated that higher expression level of *MET* in MDA-MB-231 cells than in MCF-7 cells by RT-PCR assay, which was consistent with previously studies (Fig. 2A) [17].

To confirm that the survival rate differences between MCF-7 and MDA-MB-231 cells result from the difference of *MET* expression in the two types of cells, we knocked down expression of *MET* in MDA-MB-231 cells by transfection with c-MET siRNA. After the transduction of MET siRNA, the expression of *MET* was diminished (Fig. 2B), while after treatment with NaBu, siMET transfected MDA-MB-231 cells resulted in a significantly lower survival rate compared to the NaBu treated con siRNA transfected cells (control group) (Fig. 2C). It suggested that the survival rate of con siRNA treated cells and intact cells were similar (data not shown). On the other hand, after we enhanced the expression of *MET* in MCF-7 cells (Fig. 2D), the cell survival rate was increased from 12% to 26% after treated with NaBu (Fig. 2E). Thus the oncogene *MET* helps breast cancer line survival through the treatment of NaBu. Since MDA-MB-231 cells relatively express higher *MET* level, we focused our studies on MDA-MB-231 cells in the subsequent studies.

### NaBu- resistant MDA-MB-231 cell showed cancer stem cells characteristics

**Figure 3:**
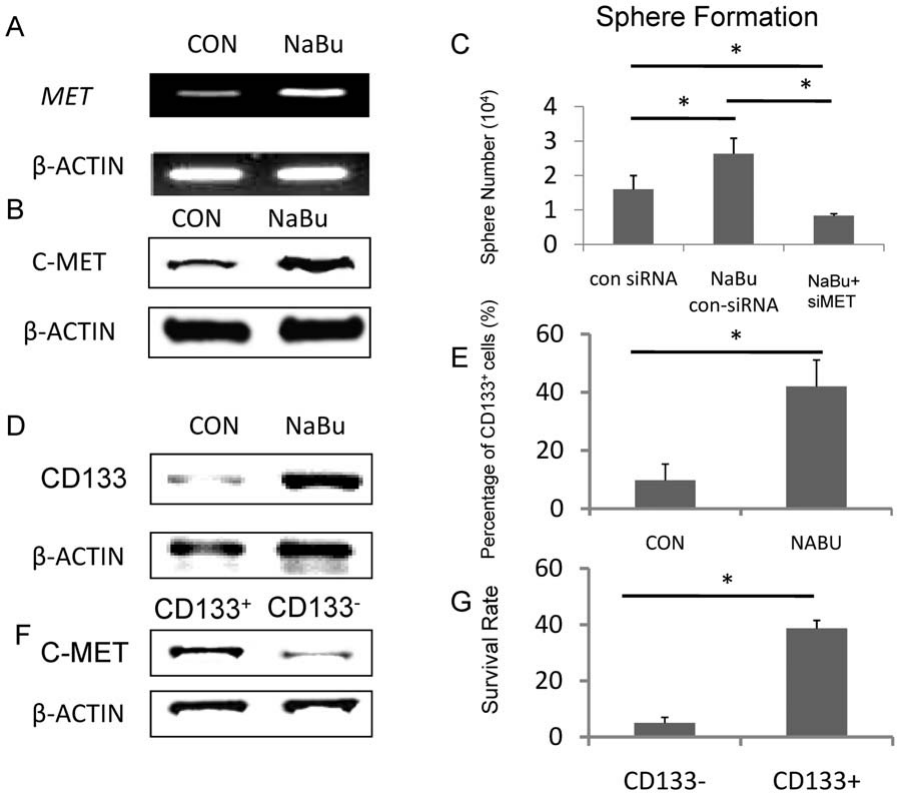
NaBu-resistant MDA-MB-231 cell showed cancer stem cells character. RT-PCR result indicate that the NaBu- resistant cell population displayes a higher expression level of *MET* (A). This was consistent with the western blot results (B). After seeding 10^5^ cells in 100 mm peridish, NaBu- resistant cells showed the strongest sphere formation capability (2.7×10^4^/dish) when compared with control siRNA treated goup(1.8×10^4^/dish)and MET siRNA treated group(0.8×10^4^/dish)(C). A higher CD133 expression level by western blot in the NaBu- resistant group was observed(D). More than 40% NaBu- resistant cells were positive for CD133 expression, however, within the control siRNA treated cells, only around 10% cell were positive for CD133(E). the CD133^+^ cells expressed higher level of c-MET compare to CD133 negtive cells(F). After treated with NaBu, cell viability of CD133^+^ cells are significantly higher than that of CD133^-^ cells number . Intact MDA-MB-231 cells are used as control without the treatment of NaBu (G). *P<0.05.

Studies have shown that c-MET is closely related to the cancer stem cell phonotype and is associated with SDF-1-CXCR4 and LIF-R-LIF axes for the trafficking of normal and malignant stem cells [10]. We wondered if the NaBu- resistant population in MDA-MB-231 cells displayed a higher cancer stem cell capability. Firstly, we examined the NaBu- resistant population with an elevated expression of *MET* compared to the control group (Fig. 3A). This was consistent with the western blot results (Fig. 3B; we mixed up figure 3B previously and all of our available data show c-MET upregulation in NABU treated cells, shown as Figure S3). Since c-MET helps the NaBu-resistant population survival as we demonstrated above, therefore, it could be concluded that NaBu selected c-MET positive cells out and enriched them in the dish.

To determine the stem cell capability of NaBu-resistant MDA-MB-231 cells, sphere formation tests were performed. Firstly, we confirmed that the con siRNA treated cells have similar sphere formation ability with intact cells. The con siRNA treated or MET siRNA treated cells were applied in this experiment. NaBu-resistant breast cancer cells showed a significantly higher sphere formation ability *in vitro* than the con siRNA treated group (Fig. 3C). As expected, the sphere formation ability of NaBu resistant MET knock down group was dramatically decreased (Fig. 3C), comparing to NaBu-resistant con siRNA treated group. These results suggested that NaBu treatment enriched the c-MET positive cancer cell population which possesses the breast cancer stem cell capability.

Afterwards, We employed CD133 for the confirmation of breast cancer stem cells character of NaBu- resistant population, since CD133 is regarded as a marker for breast cancer stem cells [18]. We obtained a higher CD133 expression level by western blot in the NaBu- resistant cell initiated sphere (Fig. 3D). Correspondently, we found that in the NaBu-resistant cell initiated spheres, 40% cells expressed CD133 positively. However, only nearly 10% cells in control cell initiated spheres presenting CD133 antigen (Fig. 3E). To elucidate whether c-MET expressed higher in CD133+ population, we tried to separate CD133+ and CD133- cells in intact MDA-MB-231 cell initiated spheres by flow cytometery. The expression of c-MET in these two groups was checked by western blot. As we expected, the CD133-positive cells expressed higher level of c-MET, compared to CD133-negative cells (Figure 3F). To testify whether the CD133-positive and the CD133-negative cells respond differently to NaBu, We separated CD133+ and CD133- cells from intact MDA-MB-231 initiated sphere. The same number (10^4^/ml) of the two fraction cells were cultured with 4mM NaBu in 100 mm dish. Two days later, NaBu was removed by changing the medium. After one day, MTT assay was performed. After treated with NaBu, cell viability of CD133^+^ cells was significantly higher than that of CD133^-^ cells number. Intact MDA-MB-231 cells are used as control without the treatment of NaBu (Fig. 3G).

### NaBu- resistant MDA-MB-231 cell possessed an increasing tumor initiation ability *in vivo*

**Figure 4:**
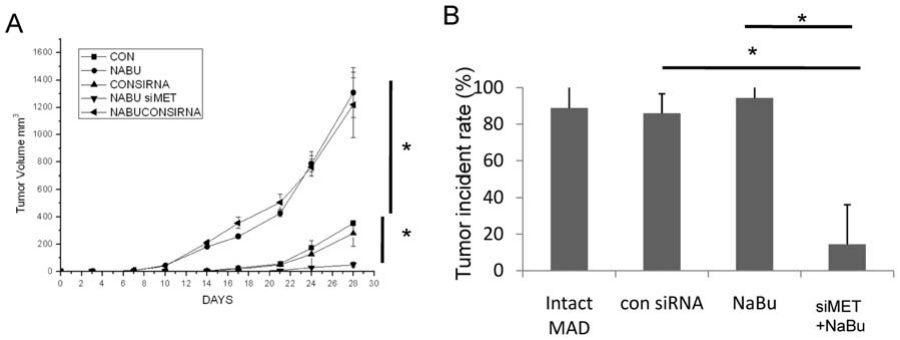
NaBu- resistant MDA-MB-231 cells displayed a high tumor initiation ability in vivo. Intact MDA-MB-231 and NaBu- resistant MDA-MB-231 cells were transplanted into NOD/SCID mouse. By the day of 28th, the tumor volume were significantly different. Moreover, tumor became palpable as early as on the 5th days in the NaBu- resistant MDA-MB-231 cell transplanted group whereas on the 10th day in the control group(A). Compared with NaBu treated intact cells, or with *MET* knock down cells, tumor volume was significantly smaller in NaBu treated *MET* knock down cell transplanted group (A). The tumor incident data were collected on the 30th day after transplanting cancer cells. The NaBu- resistant MDA-MB-231 cell transplanted group resluted in a similar tumor incident rate as the intact MDA-MB-231 group(85%). Down regulation of *MET* with a treatment of NaBu leads to decrease the tumor incident rate effectively(18%), although con siRNA teated group resulted in a similar tumor incident rate with the intact cancer cell group(83%). *P<0.05.

Since the NaBu-resistant group displayed cancer stem cells property as described above, we subsequently testified the tumor initiation ability in an animal model. The same number of NaBu-resistant cells and intact MDA-MB-231 cell was transplanted into mouse mammary fat pat. Tumors became palpable as early as on the 5th day in the mice transplanted with NaBu- resistant cells. However, in the control group, tumors became palpable on the 10th day post transplant. By the day 28th, tumor volume was significantly different between the two groups (Fig. 4A). Compare with NaBu treated intact cells, or with *MET* knock down cells, tumor volume was significantly smaller in NaBu treated *MET* knock down cell transplanted group (Fig. 4A). Meanwhile, we tried to clarify the tumorigenic effect of *MET* in the same animal model. *MET* knocked down MDA-MB-231 cells were generated before treated with 4mM NaBu for 2days. The NaBu-resistant *MET* knock down cells and con siRNA transfect cells were also transplanted into the mouse. The tumor incidence in intact MDA-MB-231 group and con siRNA group was comparable (Fig. 4B). On the 30^th^ day, the tumor incidence in NaBu- resistant *MET* knock down group was decreased to 18% as compared to con siRNA transfected cells (Fig. 4B). Again, the *MET* knock down resulted in a comparable tumor incidence rate with con siRNA treated control group (Fig. 4B). These results demonstrated that NaBu-resistant population of MAD-MB-231 cell has high tumor initiation ability, while down regulation of *MET* gene results in decreasing its tumorigenicity efficiently.

## Discussion

It have been well elaborated that NaBu exerts its tumor suppression effect on several type of cancer, such as, colon cancer, lung cancer and breast cancer [15], [19], [20], [21]. Therefore, NaBu has been regarded as a potential cancer therapy regent if one despite the short life-time of NaBu in the body [20]. In the present study, we found that, although NaBu induces majority cell death in the breast cancer cells, the minority cells that share the breast cancer stem cell characteristics may survive through NaBu selection. Those cells display higher ability of sphere formation *in vitro* as well as higher tumor incidence in animal model. Our studies suggested that the potential anti-tumor regent NaBu treatment may not be capable of removing breast cancer efficiently, since the seed of breast cancer, breast cancer stem cells, may resist from the treatment.

Our study clarified that the oncogene *MET* contributed to the NaBu- resistant effect of breast cancer cell. *MET* expressing breast cancer cell population display cancer stem cell characteristics, and studies illustrated that c-MET is closely related with highly aggressive cancer cell type. c-MET attenuates the apoptosise effect induced by NaBu in breast cancer cells. Our results demonstrated that when cancer cells after treated by the combination of NaBu and MET siRNA, decreased tumor incidence dramatically in the mouse model. However, in this study, a cell line was used as a simplified model to testify the effect of NaBu and c-MET on breast cancer. Further studies are needed to examine the reaction of breast cancer cells, especially cancer stem cells, to NaBu and c-MET in the patient, since cancer stem cells are resident in and dynamically regulated by its specific micro-environment. Based on this result and other studies, the *MET* expression level is different between the two cancer cell lines, MCF-7 and MDA-MB-231 [17]. It remains unclear about the relationship between estrogen receptor presentation and the expression of c-MET. For the c-MET low expressing cells, like MCF-7, it needs to be clarified whether another protective pathway exist to support cancer cells resisting the treatment of NaBu.

A NaBu-resistant population of breast cancer cells was identified in this study. This population of cells possesses cancer stem cells capability *in vitro* and *in vivo*. Although NaBu is regarded as a potential anti cancer substance, the endogenous expressing c-MET cancer stem cell population survived after the treatment of NaBu. A combination treatment of MET siRNA and NaBu administration can efficiently eliminate breast cancer. This study may help to develop a new strategy for breast cancer therapy.

## Materials and Methods

### Cell cultures

Human breast cancer cell MDA-MB-231 (ATCC, Manassas, VA) were maintained in Dulbecco's Modified Eagle's Medium (DMEM; Gibco BRL, Grand Island. NY) supplemented with 10% heat-inactivated fetal bovine serum (FBS, Gibco), penicillin (100 U/ml, Gibco) and streptomycin (100 µg/ml, Gibco) at 37°C in a CO2 atmosphere. NaBu (Sigma, ST. Louis, MO)was dissolved in the culture medium to the indicated concentration for treating breast cancer cells.

### MTT assay

The cell survival rate was quantified by MTT assay. After transfected with MET siRNA, cells were allowed to recover in fresh medium. One day later, medium were changed with or without NaBu(4mM). After 2 days, cells were washed in PBS and recovered in fresh medium for another two days. Sicontrol were used as siRNA transfection control. In some experiments, the intact MDA-MB-231 cells and intact MCF-7 were engaged to test the effect of NaBu on these two cell lines as indicated. MTT (50 µl of 2 mg/ml) in 1× PBS was added to each dish, and the cells were incubated for 4 hrs at 37°C. The plates were then centrifuged at 500 × g for 10 min, and DMSO (120 µl, Sigma) was added to each well and incubated 1 hr on an orbital shaker. The A570 nm was determined using an Ultra-microplate reader (ELx 808; Bio-Tek Instruments, Winooski, VT).

### Sphere- forming assays

For the colony formation assay, studies were preformed following the previous report [22].,con siRNA(Control siRNA, Santa Cruz, CA) treated MDA-MB-231 cells were treated by 4mM NaBu. After two days, cells were allowed to recover for 2days in fresh medium without NaBu. To testify the effect of c-MET on the sphere formation ability of the Nabu resistant cells, the MET siRNA (Santa Cruz) treated MDA-MB-231 cells were treated by 4mM NaBu for 2days. After recovering for 2days in fresh medium, the cells were engaged in sphere formation assay. In detail, cells were trypsinized to generate single-cell suspensions and counted by a hemocytometer. Single-cell suspensions (105 cells per 100 mm dish) were plated with an ultralow attachment surface. Three dishs were seeded for each cell line, and triplicate experiments were performed. Tumor spheres were counted and images of each well were taken on day 9. Immediately after the cells were seeded, each well was checked under the microscope to verify the sparseness of each spherical culture, and only wells containing single-cell suspension with no cell cluster were chosen for tumor-sphere counting after 9 days. Colonies of at least 60 µm in diameter (determined by using an eyepiece graticule with crossed scales) were counted on day 9 after plating. Con siRNA treated MDA-MB-231 cells were used as control group.

### Western blot analysis

Protein was extracted from the cells after culturing in the indicated conditions. After measuring the protein concentrations of homogenized lysates, 10 µg of protein extracted from cancer cells was separated by 10% SDS–PAGE and transblotted onto a polyvinylidene fluoride (PVDF) membrane. After blocking in a powdered nonfat milk solution (5% in PBS) with 0.05% Tween-20, the blot was incubated with a polyclonal rabbit anti- human Bcl-2-associated death promoter (BAD) antibody (Cell Signaling, Danvers, MA),a rabbit anti- human BAX antibody (Cell Signaling), a rabbit anti- human Bcl-2 antibody (Cell Signaling), a rabbit anti- human Cytochrome C antibody (Cell Signaling), a rabbit anti-human CD133 antibody (Cell Signaling) and a rabbit anti-human beta– actin antibody (Cell Signaling) at 1∶1000 dilutions in 5% blocking solution over night at 4°C. An anti-rabbit IgG antibody at a dilution of 1∶5000 was used as a secondary antibody. The results were detected with an enhanced chemiluminescence kit (ECL; Amersham Bioscience/GE Healthcare, Little Chalfont, UK).

### Reverse transcription (RT)- polymerase chain reaction (PCR)

Total RNA was isolated with TRIzol reagent (Invitrogen). Briefly, 500 ng of total RNA was reverse transcribed into cDNA using Avian Myeloblastosis Virus (AMV) reverse transcriptase (Takara, Ohtsu, Japan). PCR was conducted in a 20-µL reaction mixture containing 1 µL of cDNA template and 0.5 µM oligonucleotide primers. Primer sequences are:

human *MET*, forward 5′ –CATGCCGACAAGTGCAGTA-3′, reverse 5′- TCTTGCCATCATTGTCCAAC -3′,

human beta-actin, forward 5′- GACGAGGCCCAGAGCAAG- 3′, reverse 5′- ATCTCCTTCTGCATCCTGTC- 3′.

### RNA interference

Transfection of small interfering RNA (siRNA) into cells was conducted when the cells reached 70% confluence. The siRNAs of MET and a non-targeting control were purchased from Santa Cruz Biotechnology. Experiments were conducted using Dharma FECT1 (Dharmacon, Chicago, IL) as a transfection agent and siRNAs at a concentration of 100 mmol/L. Experiments were conducted according to the manufacturers' instructions.

### *MET* Retroviral Infections

Recombinant retroviruses were constructed by subcloning full-length human cDNA of Met into the retroviral expression vector pLNCX2 (Clontech, CA), which carries a neomycin phosphotransferase cassette. A retroviral expression vector carrying the cDNA of human placenta alkaline phosphatase (pLAPSN) served as a control. Experiments were conducted according to the manufacturers' instructions and previous report [23].

### Animal studies

All mouse studies were performed in accordance with protocols approved by the Animal Care and Use Committee at Southeast University (Approval ID: SEU-20100921-2). SCID mice were housed under pathogen-free conditions and were given autoclaved food and water. MDA-MB-231 cells were exposed to NaBu for 2 days. The intact MDA-MB-231cells were used as control. After determining the cell viability by trypan blue exclusion test, 2×106 cells were injected into mammary fat pad in a 100 uL volume of sterile phosphate-buffered saline. SCID mice were used as 6 weeks of age and 10 mice for each group. Tumors were measured using precision calipers twice weekly. Tumor volume was calculated at: volume  =  (length × width^2^)/2.

### Flow cytometry

After 3 washes with PBS, cells that were treated with NaBu and intact cells were detached with trypsin (Gibco) for 10 min at 37°C. For cell surface antigen phenotyping, floating and detached spindle-shaped cells were stained with fluorescent antibody CD133 (Becton–Dickinson, San Jose, CA, USA). Analyses were performed with FACS Calibur (Becton–Dickinson, NY, USA). CD133 positive cells were sorted by DAKO cytomation (DAKO, Carpinteria, CA).

### Statistical analyses

Data are presented as means ± standard error (SD) in quantitative experiments. The differences between groups were analyzed using the unpaired Student's t-test. P values <0.05 were considered significant.

## Supporting Information

Figure S1 **The expression of Bad in MDA-MB-231 and MCF-7 are different.** To confirm the a decrease in Bad expression in the MDA-MB-231 cells compared to the MCF-7 cells, triplicate studies were performed. The expression of BAD in MDA-MB-231 and MCF-7 were detected by western blot(A) .The results were quantitated and presented a significant difference in Bad expression in between MDA-MB-231 and MCF-7cells(B).
(TIF)

Figure S2 **Bad or Bax knock- down attenuated the apoptosis effect of NaBu in MDA-MB-231 cells.** The expression BAD and BAX were knock down by siBAD and siBAX respectively(A). BAD knock-dwon could moderatly prohibit the apoptosis effect of NaBu in the cells. However, BAX knockdown could protect cancer cells from the treatment of NaBu effectively as indicated by MTT assays(B).The level Cytochrome C examined by western blot was consistent with this result(C).
(TIF)

Figure S3 **NaBu resistant cells presented a higher expression of** c-MET**.** To confirm that NaBu resistant cells give a higher expression of c-MET, We did multiple experiments to confirm that c-MET expression was enhanced in NaBu resistant MDA-MB-231 cell population.
(TIF)
