# Supplementary figures and images for: c-MET Protects Breast Cancer Cells from Apoptosis Induced by Sodium Butyrate
Source: PLoS One. 2012 Jan 12;7(1):e30143. doi: 10.1371/journal.pone.0030143 (PMC3257283; doi:10.1371/journal.pone.0030143)

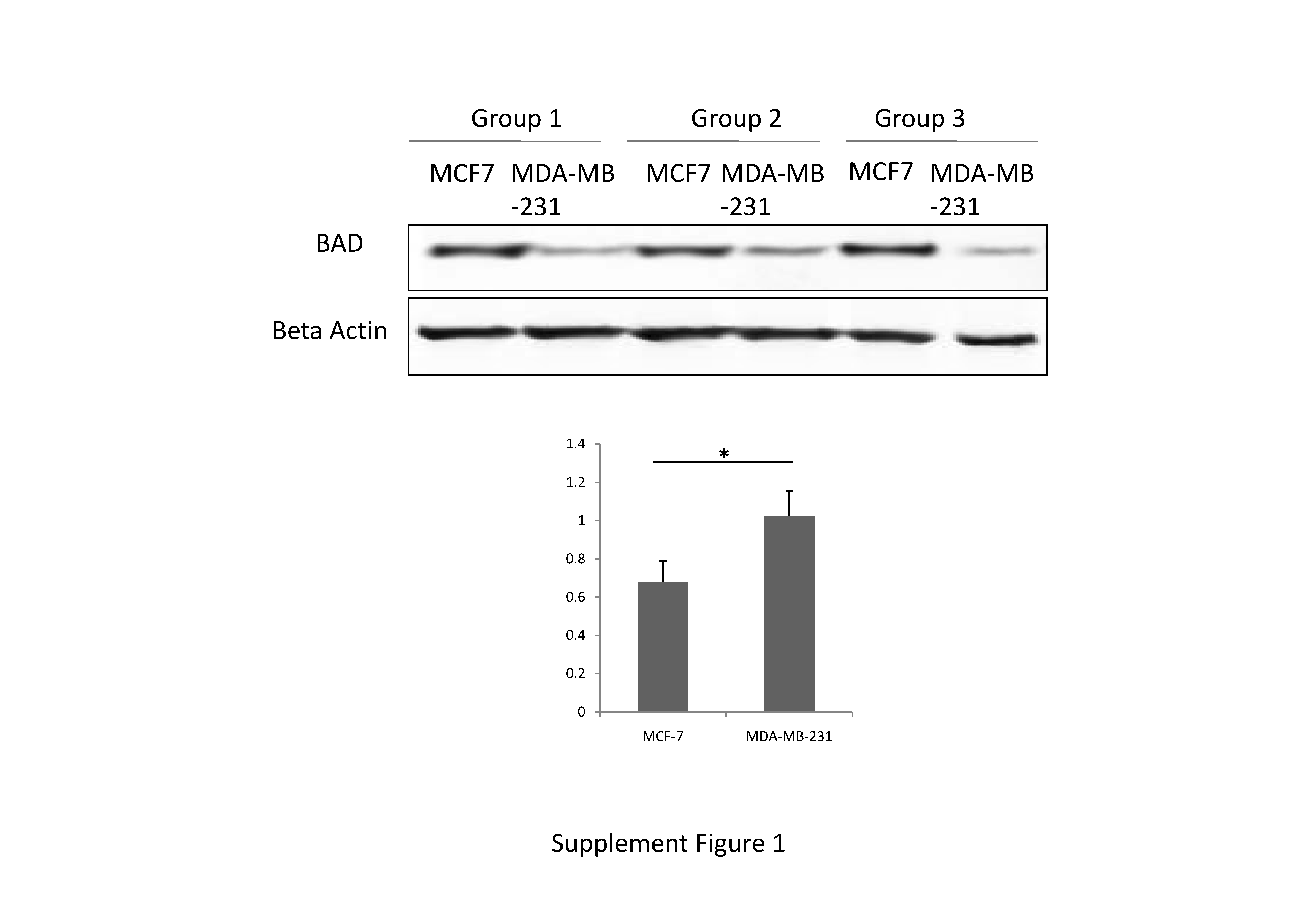

Supplement: Figure S1 — The expression of Bad in MDA-MB-231 and MCF-7 are different. To confirm the a decrease in Bad expression in the MDA-MB-231 cells compared to the MCF-7 cells, triplicate studies were performed. The expression of BAD in MDA-MB-231 and MCF-7 were detected by western blot(A) .The results were quantitated and presented a significant difference in Bad expression in between MDA-MB-231 and MCF-7cells(B). (TIF) [file pone.0030143.s001.tif]

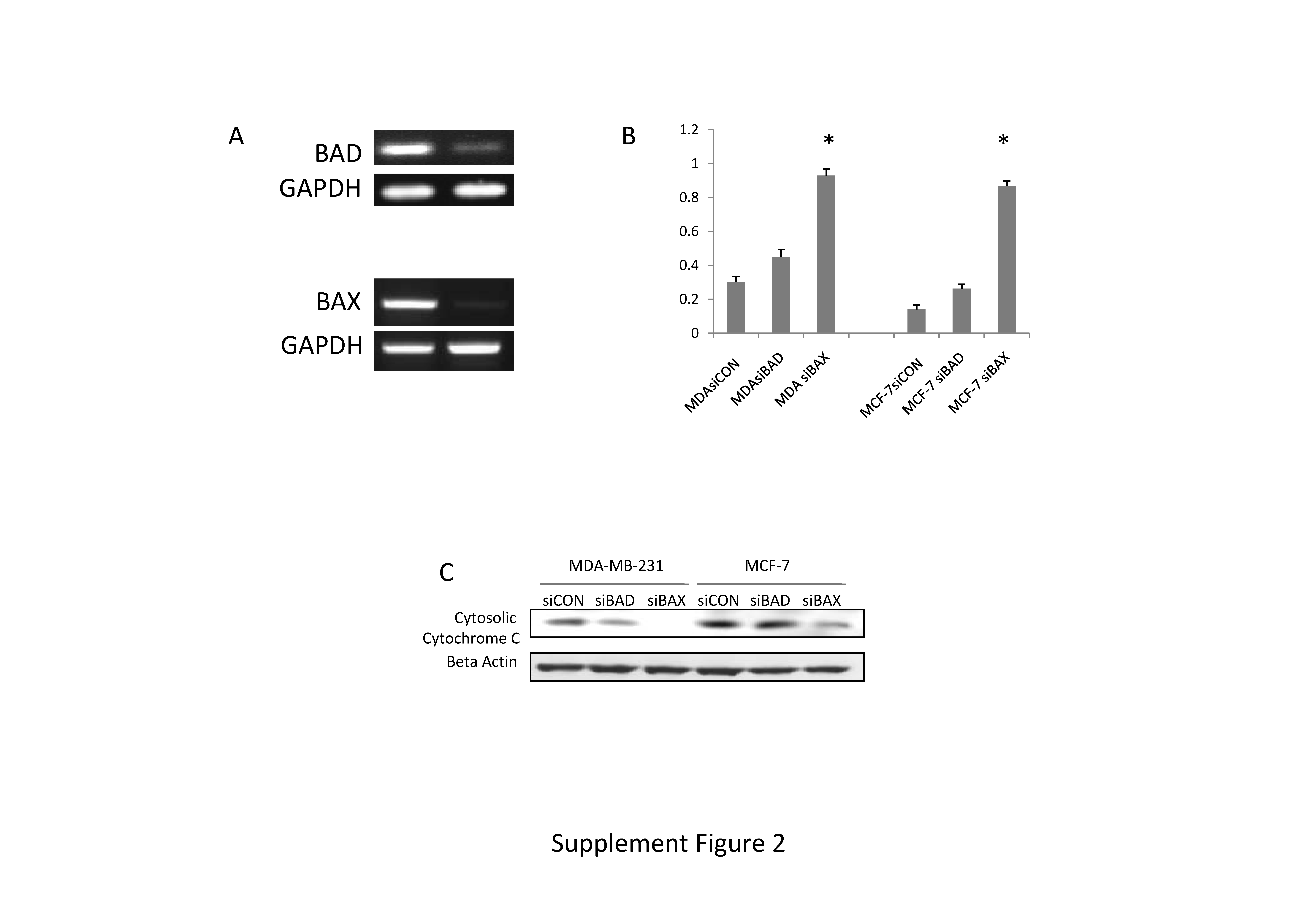

Supplement: Figure S2 — Bad or Bax knock- down attenuated the apoptosis effect of NaBu in MDA-MB-231 cells. The expression BAD and BAX were knock down by siBAD and siBAX respectively(A). BAD knock-dwon could moderatly prohibit the apoptosis effect of NaBu in the cells. However, BAX knockdown could protect cancer cells from the treatment of NaBu effectively as indicated by MTT assays(B).The level Cytochrome C examined by western blot was consistent with this result(C). (TIF) [file pone.0030143.s002.tif]

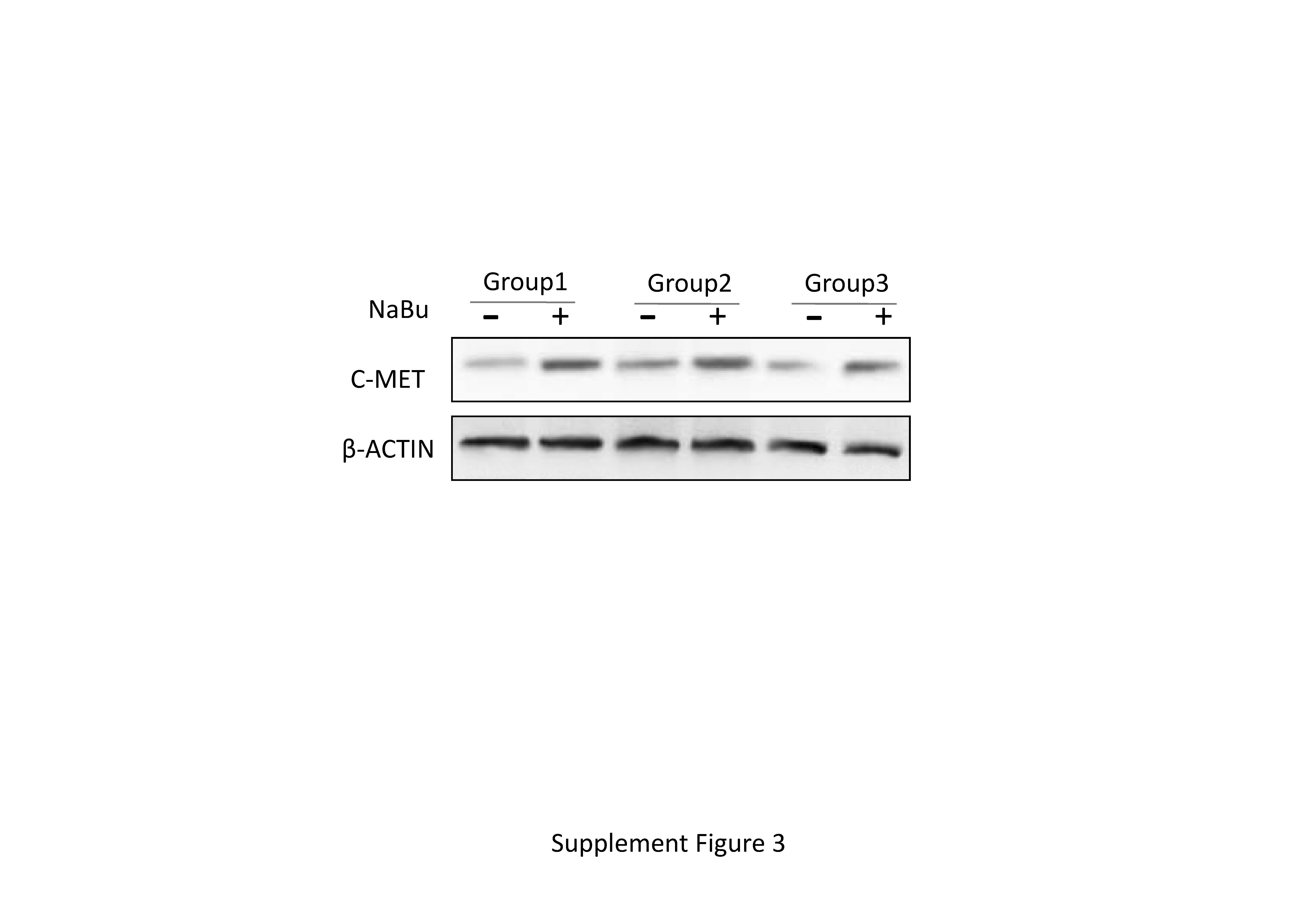

Supplement: Figure S3 — NaBu resistant cells presented a higher expression of c-MET. To confirm that NaBu resistant cells give a higher expression of c-MET, We did multiple experiments to confirm that c-MET expression was enhanced in NaBu resistant MDA-MB-231 cell population. (TIF) [file pone.0030143.s003.tif]
